# Positive Impact of Expert Reference Center Validation on Performance of Next-Generation Sequencing for Genetic Diagnosis of Autoinflammatory Diseases

**DOI:** 10.3390/jcm8101729

**Published:** 2019-10-18

**Authors:** Guilaine Boursier, Cécile Rittore, Sophie Georgin-Lavialle, Alexandre Belot, Caroline Galeotti, Eric Hachulla, Véronique Hentgen, Linda Rossi-Semerano, Guillaume Sarrabay, Isabelle Touitou

**Affiliations:** 1 Department of Medical Genetics, Rare Diseases and Personalized Medicine, CHU Montpellier, Rare and Autoinflammatory diseases unit, Univ Montpellier, 34295 Montpellier, France; 2 Department of Internal Medicine, CEREMAIA, Tenon Hospital, AP-HP, University of Pierre et Marie Curie, 75970 Paris, France; 3 Paediatric Nephrology, Rheumatology, Dermatology Unit, RAISE, HFME, HCL, Univ Lyon, 69677 Bron, France; 4 Department of Paediatric Rheumatology, CEREMAIA, Bicêtre Hospital, AP-HP, 94275 Le Kremlin-Bicêtre, France; 5 Department of Internal Medicine and Clinical Immunology, CHU Lille, University of Lille, 59037 Lille, France; 6 Department of General Pediatrics, CEREMAIA, CH Versailles, 78157 Le Chesnay, France; 7 Cellules souches, plasticité cellulaire, médecine régénératrice et immunothérapies, INSERM, Univ Montpellier, Department of Medical Genetics, Rare Diseases and Personalized Medicine, CEREMAIA, CHU Montpellier, 34295 Montpellier, Franceisabelle.touitou@inserm.fr (I.T.)

**Keywords:** autoinflammatory diseases, multidisciplinary consultation, next-generation sequencing

## Abstract

Monogenic autoinflammatory diseases (AIDs) are caused by variants in genes that regulate innate immunity. The current diagnostic performance of targeted next-generation sequencing (NGS) for AIDs is low. We assessed whether pre-analytic advice from expert clinicians could help improve NGS performance from our 4 years of experience with the sequencing of a panel of 55 AIDs genes. The study included all patients who underwent routine NGS testing between September 2014 and January 2019 at the laboratory of autoinflammatory diseases (Montpellier, France). Before March 2018, all medical requests for testing were accepted. After this time, we required validation by a reference center before NGS: the positive advice could be obtained after a face-to-face consultation with the patient or presentation of the patient’s case at a multidisciplinary staff meeting. Targeted NGS resulted in an overall 7% genetic confirmation, which is consistent with recent reports. The diagnostic performance before and after implementation of the new pre-requisite increased from 6% to 10% (*p* = 0.021). Our study demonstrated, for the first time, the beneficial effect of a two-step strategy (clinical expert advice, then genetic testing) for AIDs diagnosis and stressed the possible usefulness of the strategy in anticipation of the development of pan-genomic analyses in routine settings.

## 1. Introduction

Monogenic autoinflammatory diseases (AIDs) are characterized by variants in genes coding proteins involved in innate immunity [1]. Inflammatory episodes are either systemic, generally associated with an increase in acute phase reactants, and/or organ-specific. As the differential diagnosis of AIDs is often difficult due to overlapping clinical features, accurate genetic diagnosis is essential to initiate early and targeted treatment to prevent possible life-threatening complications of the disease. The main indication for a genetic referral is when a patient presents with clinical symptoms consistent with one or more of the AIDs [2]. Therefore, prior to any request for DNA screening, it is necessary to obtain clinical information to support the choice of screening for one or more AIDs genes. The main steps leading to genetic testing require the exclusion of infectious, autoimmune, or malignant diseases and the search for specific demographics (ethnic origin, family history) and clinical characteristics, including the evolution of inflammatory episodes (age of onset, duration of attacks, and an increase in acute phase reactants).

Since the discovery in 1997 of the first gene MEFV (Mediterranean fever (MIM 608107)), responsible for familial Mediterranean fever (FMF), more than 40 new known genetically defined AIDs have been identified, notably by high-throughput sequencing approaches (Table S1) [1,3,4,5]. The significant expansion of clinical features and genes involved in AIDs has challenged the utility of these approaches in routine diagnosis and supplanted the ‘gold standard’ Sanger sequencing. Currently, next-generation sequencing (NGS) allows for investigating multiple genes simultaneously at a manageable cost and has become the preferred method for molecular testing of AIDs [6]. Furthermore, this strategy is of particular interest for detecting low-level mosaic variants and copy-number variations (CNV) [7]. 

However, the genetic diagnosis yield of targeted panels for AIDs is still currently low (from 4% to 22% depending on how the identified genotype is interpreted) (Table S2) [8,9,10,11,12]. Because of the somewhat phenotypic similarity between the different AIDs (recurrent fever, arthralgia, abdominal pain, skin rash, etc.), formulating an initial diagnostic hypothesis is sometimes difficult, especially for physicians unfamiliar with this group of rare diseases [1]. Hence, preliminary advice from expert clinicians might help improve the performance of targeted NGS for a diagnosis of AIDs. In the 2000s, a French national program established expert structures to improve patient care and research related to rare diseases. Our reference center (CeRéMAIA) is specialized in AIDs.

Here, we presented the results of our 4 years of experience with targeted analysis of a panel of 55 known AIDs genes as a routine NGS-based strategy and compared its performance (i.e., the frequency of conclusive genetic diagnosis) before and after implementation of new pre-requisite, involving approval for this testing from a national reference center for AIDs.

## 2. Experimental Section

### 2.1. Patients and Consent

Patients referred to us for genetic diagnosis of AIDs and, if necessary, some of their relatives were fully informed and gave written consent for DNA analysis. This study included all consecutive patients who underwent routine genetic screening with the targeted NGS panel between September 2014 and January 2019. 

Before March 2018, all medical requests for NGS testing were accepted. After this date, because this permissive procedure resulted in unmanageable laboratory work overload and a disappointing rate of diagnosis confirmation, we decided to implement, with the reference centers for rare AIDs, a new decisional tree for genetic testing. For time and cost-saving, individuals with a reasonable clinical suspicion of FMF (i.e., documented biologic inflammation during at least 3 bouts of fever of unknown significance and with at-risk ethnicity) [13,14,15], deficiency of adenosine deaminase 2 (DADA2) (i.e., documented biologic inflammation during three episodes of vasculitis or stroke) [16], or functional evidence of mevalonate kinase deficiency (MKD) (i.e., elevated mevalonic aciduria during attacks) benefited from direct conventional Sanger sequencing. For the other patients, we systematically required validation by a reference center for the NGS request. A workflow describing the decisional tree for genetic testing is displayed in Figure S1. The positive advice could be obtained after a face-to-face consultation with the patient in one of these centers or presentation of the case at a monthly web-based, multidisciplinary staff meeting (MSM). The MSM included at least three reference center experts from pediatrics, internal medicine, and genetics. As per the need, other experts (e.g., dermatologists and rheumatologists) joined the MSM. To register for the MSM, physicians had to send a standardized and anonymized form containing clinical information about the patient. During MSM, prescribing doctors presented the clinical case for the patient for whom NGS was intended. After validation of the test request, they had to complete a form collecting epidemiological data and a list of clinical signs and symptoms frequently encountered in AIDs classified into six main categories (thorax, neuro-sensorial, skeletal, kidney, gastro-intestinal, and mucocutaneous). This form had to accompany the blood or DNA sample being tested.

### 2.2. Next-Generation Sequencing

All coding exons and intronic borders of 55 genes (Table S1) were screened. All the known AIDs genes at the time of the design of the panel were selected [1,17]. Some candidates’ genes presented at our biennial international congress of AIDs were also added (Table S1). The libraries were prepared from 50 ng DNA by using Nextera (Illumina, San Diego, CA, USA) or SureSelect (Agilent Technologies, Santa Clara, CA, USA) Target Enrichment Capture custom kits, according to the manufacturer’s protocols. Sequencing reactions involved the use of MiSeq or NextSeq500 equipment (Illumina, San Diego, CA, USA). Base calling, alignment, variant calling, and quality control were assessed by using SeqNext v4.3.1 (JSI medical systems, Ettenheim, Germany). The mean vertical coverage was 250× and 1235× with MiSeq and NextSeq equipment, respectively. At least 90% of the targeted regions had an average coverage depth of 40× or more.

### 2.3. Interpretation

Variants were analyzed by using standard in silico tools available on the Alamut pipeline (Interactive Biosoftware; Version 2.11): Align-Grantham variation and Grantham deviation (Align-GVGD), Sorting Intolerant From Tolerant (SIFT), Polyphen2, and MutationTaster for missense substitutions, and MaxEntScan (MES), splice-site analysis (SSF), Human Splice Finder (HSF), and neural network splice (NNSplice) for splicing defect prediction [18,19]. The variants were classified according to a 5-class score provided by the Infevers database or according to the American College of Medical Genetics and Genomics guidelines if not available [20,21]. A conclusive genetic diagnosis of AIDs was established when one likely pathogenic (class 4) or clearly pathogenic (class 5) variant was identified in a dominant condition, or two biallelic class 4 or 5 variants were identified in a recessive condition [22]. The following situations were not considered consistent with the diagnosis: one heterozygote class 4 or 5 variant or two rare variants of uncertain significance (VOUS) identified in a recessive condition; variants not segregated with the phenotype; and frequent VOUS, such as *MEFV*:p.(Glu148Gln) or *TNFRSF1A*:p.(Arg121Gln). The diagnosis was not confirmed or excluded when one rare VOUS was found for which relatives were not available for segregation analysis. We reported heterozygote status in *MEFV* because of its known dose-effect [23].

### 2.4. Statistical Analysis

We retrospectively reviewed the performance of our NGS panel and whether patients were referred by a clinician from a reference center for AIDs or their case was presented at an MSM. Clinical data were extracted from our request form for genetic diagnosis of AIDs. We used descriptive statistics, including frequencies (percentages) for categorical data and medians (Q1–Q3) for continuous data. Data were compared by two-sample Mann–Whitney rank sum, chi-square, or Fisher exact test as appropriate. *p* < 0.05 was considered statistically significant.

## 3. Results

### 3.1. Genetic Results

**Figure 1:**
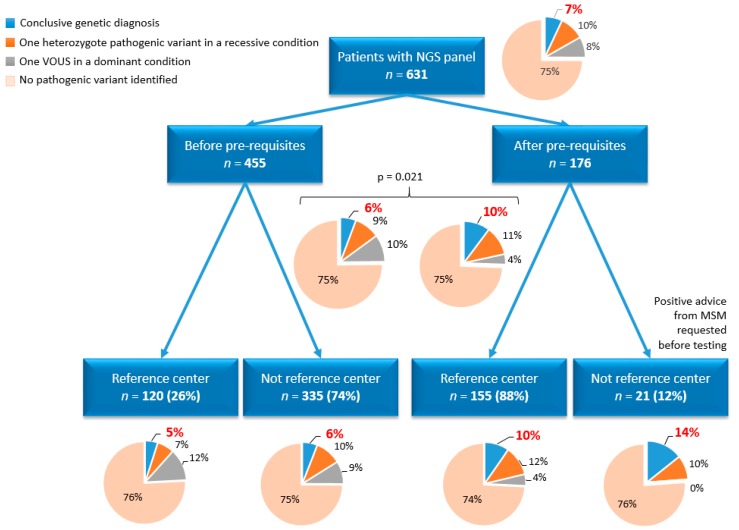
Distribution of the 631 patients who underwent testing with our panel of genes targeting 55 well-defined AIDs (autoinflammatory diseases) according to whether patients were referred by a clinician from a reference center or not (before pre-requisites, up to March 2018; after pre-requisites, after March 2018). The genetic results of NGS (next-generation sequencing) testing are displayed for each group of patients. MSM, multidisciplinary staff meeting; VOUS, variant of uncertain significance.

**Figure 2:**
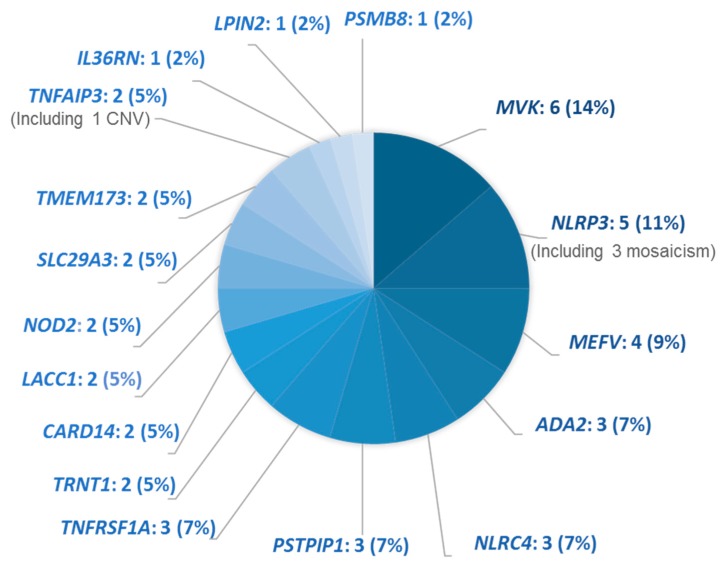
Distribution of the mutated genes identified in patients with a conclusive genetic diagnosis of hereditary AIDs. Data are a number of patients (percentage). CNV: Copy Number Variant

Figure 1 shows the distribution of the 631 patients included in this study, depending on whether they were referred before or after implementation of the new pre-requisite and by a clinician from a reference center or not. Overall, 63% (400/631) of the patients were <18 years old at referral, with no significant differences across the groups. Class 4 or 5 variants accounted for 70% (124/176) of the variants and were detected in only 30/55 genes. The most recurrent pathogenic variants were *MVK*:p.(Val377Ile), *MEFV*:p.(Met694Val), and *RNASEH2B*:p.(Ala177Thr). At least one variant was reported in one-quarter of patients (158/631). The yield of conclusive genetic diagnosis with this NGS strategy was 7% over this 4-year period (Figure 1). The genes for which we identified the pathogenic variants are depicted in Figure 2, with the most frequent in decreasing order: *MVK* (*n* = 6), *NLRP3* (*n* = 5), and *MEFV* (*n* = 4). 

### 3.2. Positive Impact of the New Pre-Requisite for NGS Testing on the Frequency of Conclusive Genetic Diagnosis

The origin of the requests for NGS considerably switched upon the implementation of the new pre-requisite for NGS (i.e., validation of each prescription by a reference center for AIDs (Figure 1)). Before this date, only one-quarter of the requests came from a reference center. After this time, one or more expert clinicians examined all patients’ records.

The diagnostic performance of our NGS panel almost doubled after March 2018, from 6% to 10% (*p* = 0.021) (Figure 1). We observed improvement when we compared requests from the subset of clinicians, whether they were from a reference center (5% vs. 10%, *p* = 0.029) or not (6% vs. 14%, NS), which supports the positive effect of the pre-requisite. The lack of significance of the subset of clinicians not from a reference center is most likely due to the considerable reduction in a number of direct requests by clinicians not from reference centers since we imposed their validation by an MSM.

### 3.3. Epidemiological and Clinical Data of the 44 Patients with a Conclusive Genetic Diagnosis

**Figure 3:**
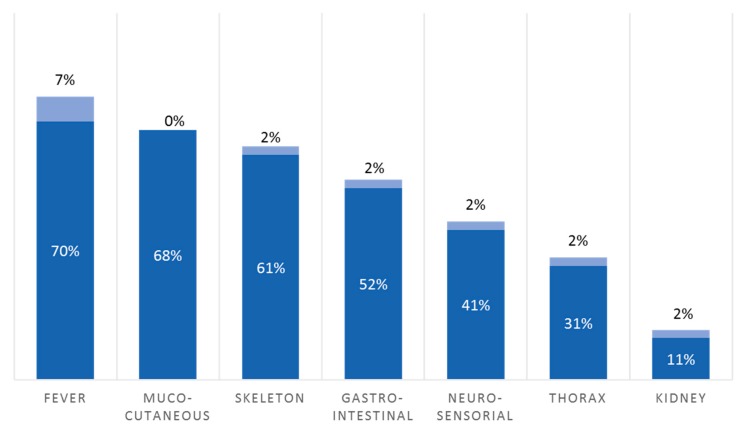
Main symptoms described in the 44 patients with a conclusive genetic diagnosis (dark blue). The lack of information regarding symptoms of each category is in light blue.

The age of onset was pediatric for all but one patient (median 18 months; range: neonatal to 53 years old). The 53-year-old patient exhibited a class 4 somatic variant (c.1708T>A, p.(Tyr570Asn), 24% mosaicism) consistent with her *NLRP3*-associated AID phenotype. Consanguinity was documented in seven of the 10 patients in whom we identified a homozygous pathogenic variant in a gene responsible for a recessive condition. The main symptoms reported are described in Figure 3. Detailed signs and symptoms included in the clinical report form were (1) fever; (2) mucocutaneous: oral aphthosis, erythema nodosum, folliculitis, lipodystrophy, maculopapules, necrosis, edema, pseudo-erysipelas, psoriasis, pyoderma, tenosynovitis, urticaria, and vasculitis; (3) skeleton: arthralgia, arthritis, and degenerative arthropathy; (4) gastro-intestinal: abdominal pain, diarrhea, vomiting, and hepatic cytolysis; (5) neuro-sensorial: stroke, conjunctivitis, epilepsy, deafness, uveitis, and papillitis; (6) thorax: interstitial pneumopathy and pericarditis; and (7) kidney: amyloidosis and proteinuria. Other common symptoms, such as lymphadenopathy and hepato-splenomegaly, or specific symptoms, such as retroperitoneal fibrosis (*SLC29A3*-associated histiocytosis-lymphadenopathy–plus syndrome) or sideroblastic anemia (*TRNT1*-associated sideroblastic anemia with B-cell immunodeficiency, periodic fever, and developmental delay), were also reported.

## 4. Discussion

Recognizing patients with monogenic AIDs is difficult because their clinical spectrum may range from very mild inflammatory episodes to severe chronic disease. Moreover, the disease features significantly overlap [1]. Our targeted NGS approach resulted in genetic confirmation in a relatively small proportion (7%) of patients. This finding is consistent with two 2019 reports by Karacan et al. and Papa et al., who used recent guidelines for the definition of positive diagnosis as we did [10,12]. Three previous studies using a broader definition claimed 19%, 20%, and 22% diagnostic yield [8,9,11]. In those reports, for example, frequent variants, such as p.(Glu148Gln) (*MEFV*), p.(Arg121Gln) (*TNFRSF1A*), or p.(Val198Met) (*NLRP3*), and all known susceptibility factors for inflammation were considered consistent with the diagnosis [8,9,11,21,22]. Moreover, our definition was more stringent than these four previous studies because we considered that we could not conclude on the diagnosis when the parent’s DNA was lacking segregation analyses of heterozygous rare VOUS.

The low performance of the AIDs panel might also be explained by our study design that did not include most of the patients with features of FMF, MKD, and DADA2 because they were screened by Sanger sequencing. Still, the genes *MEFV*, *MVK,* and *ADA2* remained among the four most frequently involved in the conclusive diagnoses of our study. This observation possibly stems from a better detection by NGS of the new molecular mechanisms involved in AIDs (mosaicism, CNVs) and better information on these three diseases because they are among the best-described AIDs [21,24]. In addition, FMF is now recognized as a disease caused by gain-of-function variants with a dosage effect rather than a purely recessive condition [23]. For one-third of the patients with one heterozygous pathogenic variant in a recessive condition, the gene was *MEFV*. If we had included heterozygous carriers of a *MEFV* pathogenic variant, the genetic diagnosis yield would have improved up to 10% (Figure 2).

In an attempt to find a cause that could explain the low rate of genetic confirmation using panels in the patients referred to us, we wondered whether validation of the request by a specialized reference center could improve this performance. The success of the NGS approach was suggested to rely on its use in the context of a highly specialized clinical service for patients with AIDs [8]. National reference centers and networks cover all the expertise necessary for patient care and operational management and efficiency [25]. We established a pre-requisite for a systematic evaluation of undifferentiated AIDs patients by clinicians from reference centers. It is possible that many doctors from non-reference centers could not find the time to present their patients to MSM. This certainly accounts for the decrease in the proportion of patients referred by the non-reference centers. However, our study showed that this strategy resulted in a better clinical filter for ordering genetic tests and almost doubled the performance of our NGS strategy. Also, our reference center recently offered a diagnostic checklist to guide practitioners who may suspect an AID for ordering genetic tests [26]. In our patients with a conclusive diagnosis of AID, the main features identified were mucocutaneous, articular, and gastro-intestinal. This result reflects daily practice because the cutaneous/mucosal, digestive tract, and musculoskeletal systems are the three most common systems involved in AIDs [26].

We cannot rule out a relative underestimation of the potential of NGS panels for AIDs in general. Indeed, because of our study design, some of the patients or their relatives were not available for NGS or segregation analysis. However, our aim was not to evaluate the exact performance of NGS but to find ways to improve it. In addition to the successful clinical option we demonstrated here, it is easy to predict the upcoming discovery of new genes or molecular mechanisms involved in AIDs that will continue to revolutionize our routine genetic diagnosis [17,27]. Recent inputs of the literature advocate closer interactions between clinicians and geneticists in the era of whole-genome sequencing. Among them are the identification of genetic modifiers, affecting the penetrance of homozygous pathogenic *MVK*:p.(Val377Ile) variant [28]; the lack of detection of pathogenic variants in patients with a clear given phenotype, such as pathogenic canonical splice site variants not covered by Sanger and panel approaches (e.g., *ADA2*:c.-47+2T>C) [29]; or large chromosomal rearrangements, encompassing several genes in patients with complex phenotypes [30]. In those circumstances, an MSM could help find the best appropriate strategy in accordance with the patient’s phenotype.

The role of the reference centers is to determine whether or not the clinical/anamnesis/genealogical tree is compatible with a known AID. If compatibility does not seem to be the case, the role is to evaluate the potential added value to perform the NGS. If this value appears to be low, the reference center recommends that no NGS be conducted in discussion with geneticists. Thus, this discussion decreases the number of individuals whose phenotype is not typically enough.

Our results confirmed the currently low performance of the use of NGS panels for the genetic diagnosis of AIDs [10]. Our targeted NGS approach resulted in genetic confirmation in only 7% of patients in our 4 years of experience. However, for the first time, we highlighted the beneficial effect of a referral to expert clinicians on the frequency of conclusive genetic diagnosis of a large panel of genes targeting well-defined AIDs. Altogether, our study stressed the usefulness of this two-step approach (clinical filter, then genetic testing), both currently and in anticipation of the development of pan-genomic exome and genome analyses in routine settings.

## Supplementary Materials

The following are available online at https://www.mdpi.com/2077-0383/8/10/1729/s1, Table S1. List of the 55 genes and transcripts used for variant annotation and nomenclature included in our next-generation sequencing targeted panel and association with OMIM diseases. Table S2. Genetic diagnosis yield of targeted panels for AIDs reported in the literature. Figure S1. Workflow reporting the decisional tree for genetic testing.
