# Supplementary material for: Positive Impact of Expert Reference Center Validation on Performance of Next-Generation Sequencing for Genetic Diagnosis of Autoinflammatory Diseases
Source: J Clin Med. 2019 Oct 18;8(10):1729. doi: 10.3390/jcm8101729 (PMC6832712; doi:10.3390/jcm8101729)
Supplement: Supplementary file 1 [file jcm-08-01729-s001.pdf]

Journal of
*Clinical Medicine*

MDPI

*Original Article*

# Postitive impact of impact of expert reference centre validation on performance of next-generation sequencing for genetic diagnosis of autoinflammatory diseases

**Guilaine Boursier[1]*, Cécile Rittore[1], Sophie Georgin-Lavialle[2], Alexandre Belot[3], Caroline Galeotti[4], Eric Hachulla[5], Véronique Hentgen[6], Linda Rossi-Semerano[4], Guillaume Sarrabay[7] and and Isabelle Touitou[7]**

**Supplementary Materials:** The following are available online at www.mdpi.com/xxx/s1, Table S1: List of the 55 genes and transcripts used for variant annotation and nomenclature included in our next-generation sequencing targeted panel and association with OMIM diseases. Table S2: Genetic diagnosis yield of targeted panels for AIDs reported in the literature. Figure S1. Workflow reporting the decisional tree for genetic testing.

**Table S1.** List of the 55 genes and transcripts used for variant annotation and nomenclature included in our next-generation sequencing targeted panel and association with OMIM diseases.

| GENETICS | | | PHENOTYPE | | | | | |
|---|---|---|---|---|---|---|---|---|
| **Gene (locus)** | **RefSeq transcript** | **OMIM** | **Inheritance** | **Disease** | **Name of disease** | **OMIM** | **Aliases** | **Name of aliases** |
| ACP5 (19p13.2) | NM_001111035.2 | 171640 | Recessive | SPENCDI | Spondyloenchondrodysplasia With Immune Dysregulation | 607944 | | |
| ADA2 (22q11.1) | NM_001282225.1 | 607575 | Recessive | VAIHS | Vasculitis, Autoinflammation, Immunodeficiency, And Hematologic Defects Syndrome | 615688 | DADA2 | Deficiency of adenosine deaminase 2 |
| | | | Recessive | SNDNS | Sneddon Syndrome | 182410 | | |
| | | | Recessive | PAN | Polyarteritis Nodosa, Childhood-Onset | | | |
| ADAM17 (2p25.1 ) | NM_003183 | 603639 | Recessive | NISBD | Inflammatory Skin And Bowel Disease, Neonatal | 614328 | | |
| ADAR (1q21.3 ) | NM_001111 | 146920 | Recessive | AGS6 | Aicardi-Goutieres Syndrome 6 | 615010 | | |
| | | | Dominant | DSH | Dyschromatosis Symmetrica Hereditaria | 127400 | | |
| AP1S3 (2q36.1 ) | NM_001039569.1 | 615781 | Dominant | PSORS15 | Psoriasis 15, Pustular, Susceptibility To | 616106 | | |
| CARD14 (17q25.3) | NM_024110.4 | 607211 | Dominant | PSOR2 | Psoriasis 2 | 602723 | CAMPS | CARD14-mediated pustular psoriasis |
| | | | Dominant | PRP | Pityriasis Rubra Pilaris | 173200 | | |
| CASP1 (11q22.3) | NM_001257118 | 147678 | Candidate | | | | | |
| COPA (1q23.2) | NM_001098398.1 | 601924 | Dominant | AILJK | Autoimmune Interstitial Lung, Joint, And Kidney Disease | 616414 | | |
| DDX58 (9p21.1) | NM_014314 | 609631 | Dominant | SGMRT2 | Singleton-Merten Syndrome 2 | 616298 | | |

| | | | | | | | | |
|---|---|---|---|---|---|---|---|---|
| FAS (10q23.31) | NM_000043.5 | 134637 | Dominant/ Recessive | ALPS | Autoimmune Lymphoproliferative Syndrome | 601859 | | |
| FASLG (1q24.3) | NM_000639.2 | 134638 | Recessive | ALPS | Autoimmune Lymphoproliferative Syndrome | 601859 | | |
| FBLIM1 (1p36.21) | NM_017556.3 | 607747 | Candidate (AR) | CRMO | Chronic Recurrent Multifocal Osteomyelitis | | | |
| IFIH1 (2q24.2) | NM_022168.3 | 606951 | Dominant | AGS7 | Aicardi-Goutieres Syndrome 7 | 615846 | | |
| | | | Dominant | SGMRT1 | Singleton-Merten Syndrome 1 | 182250 | | |
| IL10 (1q32.1) | NM_000572.2 | 124092 | Candidate | IL10 deficiency | | | | |
| IL10RA (11q23.3) | NM_001558.3 | 146933 | Recessive | IBD28 | Inflammatory Bowel Disease 28, Autosomal Recessive | 613148 | | |
| IL10RB (21q22.11) | NM_000628.4 | 123889 | Recessive | IBD25 | Inflammatory Bowel Disease 25, Autosomal Recessive | 612567 | | |
| IL1RN (2q14.1) | NM_173841.2 | 147679 | Recessive | OMPP | Osteomyelitis, Sterile Multifocal, With Periostitis And Pustulosis | 612852 | DIRA | Deficiency of IL1 Receptor Antagonist |
| IL36RN (2q14.1) | NM_173170.1 | 605507 | Recessive | PSORP14 | Pustular Psoriasis, Generalized | 614204 | DITRA | Deficiency of IL36 Receptor Antagonist |
| LACC1 (13q14.11) | NM_001128303.1 | 613409 | Candidate (AR) | SJIA | Systemic Juvenile Inflammatory Arthritis | | | |
| LPIN2 (18p11.31) | NM_014646.2 | 605519 | Recessive | MJDS | Majeed Syndrome | 609628 | LPIN2-CNO | LPIN2-Chronic non-bacterial osteomyelitis |
| MDFIC (7q31.1-q31.2) | NM_199072.4 | 614511 | Candidate | | | | | |
| MEFV (16p13.3) | NM_000243.2 | 608107 | Recessive | FMF | Familial Mediterranean Fever | 249100 | | |
| | | | Dominant | FMF | Familial Mediterranean Fever, Autosomal Dominant | 134610 | | |

| | | | | | | | | |
|---|---|---|---|---|---|---|---|---|
| | | | Dominant | PFAD | Periodic Fever With Autoinflammatory Disease | | | |
| | | | Dominant | PAAND | Pyrin-Associated Autoinflammation With Neutrophilic Dermatosis | | | |
| MVK (12q24.11) | NM_000431.3 | 251170 | Recessive | MEVA | Mevalonic Aciduria | 610377 | MKD | Severe Mevalonate kinase deficiency |
| | | | | HIDS | Hyper-Igd Syndrome | 260920 | MKD | Mild Mevalonate kinase deficiency |
| NCSTN (1q23.2) | NM_015331.2 | 605254 | Dominant | ACNINV1 | Acne Inversa, Familial, 1 | 142690 | PASH | Pyoderma gangrenosum acne suppurative hidradenitis |
| NLRC4 (2p22.3) | NM_021209.4 | 606831 | Dominant | AIFEC | Autoinflammation With Infantile Enterocolitis | 616050 | | |
| | | | | FCAS4 | Familial Cold Autoinflammatory Syndrome 4 | 616115 | | |
| NLRP1 (17p13.2) | NM_033004.3 | 606636 | Dominant/ Recessive | AIADK | Autoinflammation With Arthritis And Dyskeratosis; | 617388 | NAIAD | NLRP1-associated autoinflammation with arthritis and dyskeratosis |
| NLRP12 (19q13.42) | NM_144687.3 | 609648 | Dominant | FCAS2 | Familial Cold Autoinflammatory Syndrome 2 | 611762 | | |
| NLRP3 (1q44) | NM_001243133.1 | 606416 | Dominant | CINCA | Cinca Syndrome | 607115 | NOMID | Neonatal onset multisystem inflammatory disease |
| | | | | | | | Severe NLRP3-AID | NLRP3-associated autoinflammatory disease (NLRP3-AID) |
| | | | Dominant | MWS | Muckle-Wells Syndrome | 191900 | Moderate NLRP3-AID | |
| | | | Dominant | FCAS1 | Familial Cold Autoinflammatory Syndrome 1 | 120100 | Mild NLRP3-AID | |
| NOD2 (16q12.1) | NM_022162.2 | 605956 | Dominant | Blau | Blau Syndrome | 186580 | | NOD2-associated granulomatous disease |

| | | | | | | | | |
|---|---|---|---|---|---|---|---|---|
| OTULIN (5p15.2) | NM_138348 | 615712 | Recessive | AIPDS | Autoinflammation, Panniculitis, And Dermatosis Syndrome | 617099 | | |
| PLCG2 (16q23.3) | NM_002661.4 | 600220 | Dominant | APLAID | Autoinflammation, Antibody Deficiency, And Immune Dysregulation, Plcg2-Associated | 614878 | | |
| | | | | FCAS3 | Familial Cold Autoinflammatory Syndrome 3 | 614468 | PLAID | PLCG2 associated antibody deficiency and immune dysregulation |
| POMP (13q12.3) | NM_015932.5 | 613386 | Recessive | KLICK | Keratosis Linearis With Ichthyosis Congenita And Sclerosing Keratoderma | 601952 | | |
| | | | Dominant | PRAAS2 | Proteasome-Associated Autoinflammatory Syndrome 2 | 618048 | CANDLE | Chronic Atypical Neutrophilic Dermatosis with Lipodystrophy and Elevated temperature |
| PSENEN (19q13.12) | NM_172341.2 | 607632 | Dominant | ACNINV2 | Acne Inversa, Familial, 2 | 613736 | | |
| PSMA3 (14q23.1) | NM_002788.3 | 176843 | Candidate (AR) | PRAAS | | | | |
| PSMB4 (1q21.3) | NM_002796.2 | 602177 | Recessive | PRAAS3 | Proteasome-Associated Autoinflammatory Syndrome 3 | 617591 | CANDLE | Chronic Atypical Neutrophilic Dermatosis with Lipodystrophy and Elevated temperature |
| PSMB8 (6p21.32) | NM_148919.3 | 177046 | Recessive | PRAAS1 | Proteasome-Associated Autoinflammatory Syndrome 1 | 256040 | CANDLE | Chronic Atypical Neutrophilic Dermatosis with Lipodystrophy and Elevated temperature |
| PSMB9 (6p21.32) | NM_002800.4 | 177045 | Recessive | PRAAS3 | Proteasome-Associated Autoinflammatory Syndrome 3 | 617591 | CANDLE | Chronic Atypical Neutrophilic Dermatosis with Lipodystrophy and Elevated temperature |
| PSMG2 (18p11.21) | NM_020232.4 | 609702 | Candidate (AR) | PRAAS | Proteasome Associated Autoinflammatory Syndrome | | | |
| PSTPIP1 (15q24.3) | NM_003978.4 | 606347 | Dominant | PAPA | Pyogenic Sterile Arthritis, Pyoderma Gangrenosum, And Acne | 604416 | PAPA | PSTPIP1-associated arthritis, pyoderma gangrenosum and acne |
| PYCARD (16p11.2) | NM_013258.4 | 606838 | Candidate | | | | | |
| RBCK1 (20p13) | NM_031229.3 | 610924 | Recessive | PGBM1 | Polyglucosan Body Myopathy 1 With Or Without Immunodeficiency | 615895 | PBMEI | Polyglucosan Body Myopathy, Early-Onset, With or Without Immunodeficiency |

| | | | | | | | | |
|---|---|---|---|---|---|---|---|---|
| RNASEH2A (19p13.13) | NM_006397.2 | 606034 | Recessive | AGS4 | Aicardi-Goutieres Syndrome 4 | 610333 | | |
| RNASEH2B (13q14.3) | NM_024570.3 | 610326 | Recessive | AGS2 | Aicardi-Goutieres Syndrome 2 | 610181 | | |
| RNASEH2C (11q13.1) | NM_032193.3 | 610330 | Recessive | AGS3 | Aicardi-Goutieres Syndrome 3 | 610329 | | |
| RNF31 (14q12) | NM_017999.4 | 612487 | Candidate (AR) | HOIP Deficiency | | | | |
| SAMHD1 (20q11.23) | NM_015474.3 | 606754 | Recessive | CHBL2 | Chilblain Lupus 2 | 614415 | | |
| | | | | AGS5 | Aicardi-Goutieres Syndrome 5 | 612952 | | |
| SERPING1 (11q12.1) | NM_000062.2 | 606860 | Dominant | HAE1 | Angioedema, Hereditary, Type I | 106100 | | |
| SH3BP2 (4p16.3) | NM_003023.4 | 602104 | Dominant | | Cherubism | 118400 | SDCM | SH3BP2 deficiency with multilocular cystic disease of the mandibles |
| SLC29A3 (10q22.1) | NM_018344.5 | 612373 | Recessive | H syndrome | Histiocytosis-Lymphadenopathy Plus Syndrome | 602782 | | |
| TMEM173 (5q31.2) | NM_198282.3 | 612374 | Dominant | SAVI | Sting-Associated Vasculopathy, Infantile-Onset | 615934 | | |
| TNFAIP3 (6q23.3) | NM_001270508.1 | 191163 | Dominant | AISBL | Autoinflammatory Syndrome, Familial, Behcet-Like | 616744 | HA20 | Deficiency in HA20 protein |
| TNFRSF11A (18q21.33) | NM_003839.3 | 603499 | Recessive | OPTB7 | Osteopetrosis, Autosomal Recessive 7 | 612301 | TRAPS11 | TNFRSF11A-associated hereditary fever disease |
| | | | Dominant | FEO | Familial Expansile Osteolysis | 174810 | | |
| TNFRSF1A (12p13.31) | NM_001065.3 | 191190 | Dominant | | Periodic Fever, Familial, Autosomal Dominant | 142680 | TRAPS | TNF receptor-associated periodic fever syndrome |
| TNFRSF9 (1p36.23) | NM_001561 | 602250 | Candidate | | | | | |

| | | | | | | |
|---|---|---|---|---|---|---|
| TREX1 (3p21.31) | NM_016381.5 | 606609 | Dominant/ Recessive | CHBL1 | Chilblain Lupus 1 | 610448 |
| | | | Dominant | AGS1 | Aicardi-Goutieres Syndrome 1 | 225750 |
| | | | Dominant | RVCL | Vasculopathy, Retinal, With Cerebral Leukodystrophy | 192315 |
| TRNT1 (3p26.2) | NM_182916.2 | 612907 | Recessive | RPEM | Retinitis Pigmentosa And Erythrocytic Microcytosis | 616959 |
| | | | Recessive | SIFD | Sideroblastic Anemia With B-Cell Immunodeficiency, Periodic Fevers, And Developmental Delay | 616084 |

AR, Autosomal recessive. OMIM, Online Mendelian Inheritance in Man, OMIM®. McKusick-Nathans Institute of Genetic Medicine, Johns Hopkins University (Baltimore, MD), {13/09/2019}. World Wide Web URL: https://omim.org/

**Table S2**. Genetic diagnosis yield of targeted panels for AIDs reported in the literature.

| Reference | No of AIDs genes in the panel | Library enrichment method | NGS equipment | Software for raw data analysis | Variant pathogenicity assessment | Inclusion of frequent VOUS | Segregation analysis | Coverage, depth of reads | No. of patients tested | No. of patients with genetic confirmation | Genetic diagnosis yield |
|---|---|---|---|---|---|---|---|---|---|---|---|
| Omoyinmi et al., 2017 | 32 | Capture (QXT, Agilent technologies) | MiSeq (Illumina) | Galaxy, in-house pipeline and Agilent SureCall v3.5.1.46 | ACGS 2013 guidelines | Yes | Yes if available | 97%, >30X | 50 | 11 | 22% |
| Nakayama et al., 2017 | 12 | Amplification (Multiplex PCR, Takara) | MiSeq (Illumina) | Blat (aligner) and Sommelier (variant caller) | ND | Yes | ND | 90%, >20X | 108 | 22 | 20% |
| Ozyilmaz et al., 2019 | 3 | NEXTflex Periodic Fever-1 NGS Amplicon Panel (Bioo Scientific) | MiSeq (Illumina) | "SEQ" variant analysis (Genomize) | ND | Yes | Yes if available | ND | 64 | 12 | 19% |
| Karacan et al., 2019 | 15 | Amplification (Ion AmpliSeq, Thermo Fisher Scientific) | Ion S5 (Thermo Fisher Scientific) | Torrent Suite 5.4.0 (Life Technologies) | Frequencies, HGMD, ClinVar, Infevers | No | ND | 95%, >50X | 196 | 14 | 7% |
| Papa et al., 2019 | 41 | Amplification (Ion AmpliSeq, Thermo Fisher Scientific) | Ion PGM™ (Thermo Fisher Scientific) | Ion Reporter™ 5.0 (Thermo Fisher Scientific) | In-house criteria (Frequencies, *in silico* software tools, CADD score, ClinVar) | No | Yes if available | 4 amplicons at <10X analyzed by Sanger sequencing | 50 | 2 | 4% |

ND, not determined; ACGS, Association for Clinical Genomic Science; HGMD, Human Gene Mutation Database.

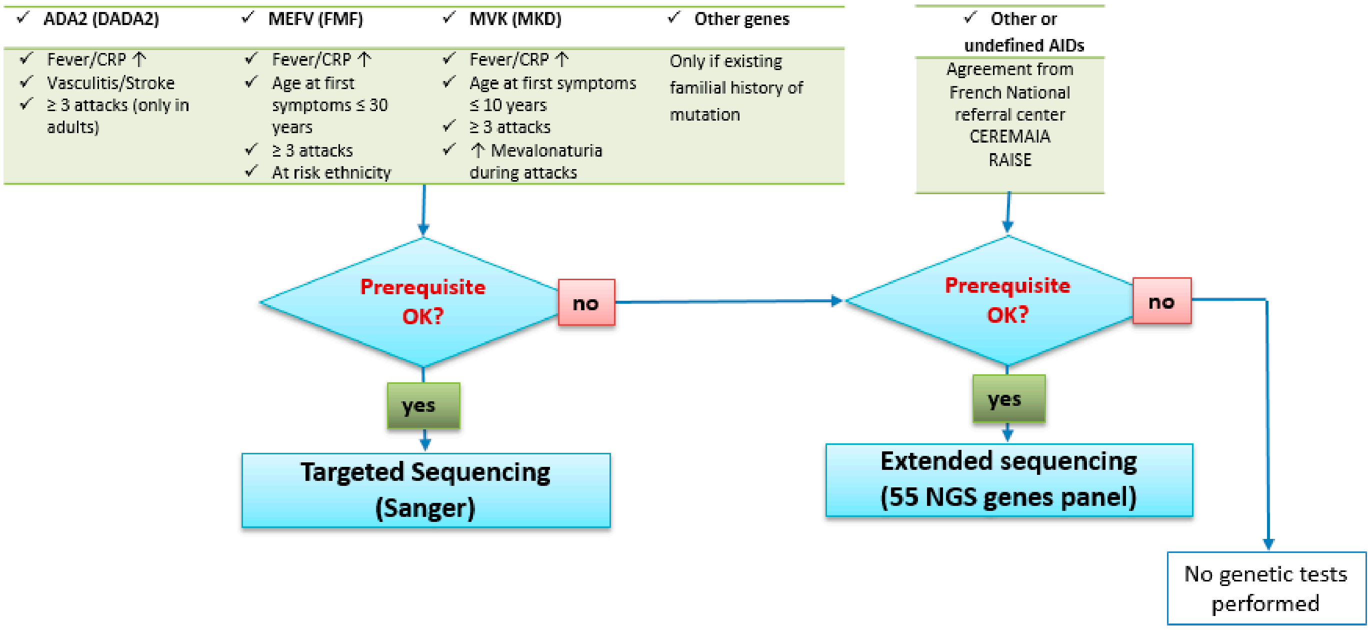


**Figure S1**. Workflow reporting the decisional tree for genetic testing. CEREMAIA, Reference centre for AIDs; CRP, C-reactive protein; RAISE, Reference centre for juvenile arthritis and pediatric rare autoimmune diseases.
